# Alternative Mechanisms for Talin to Mediate Integrin Function

**DOI:** 10.1016/j.cub.2015.01.043

**Published:** 2015-03-30

**Authors:** Benjamin Klapholz, Samantha L. Herbert, Jutta Wellmann, Robert Johnson, Maddy Parsons, Nicholas H. Brown

**Affiliations:** 1 The Gurdon Institute and Department of Physiology, Development and Neuroscience, University of Cambridge, Tennis Court Road, Cambridge CB2 1QN, UK; 2 Randall Division of Cell and Molecular Biophysics, King’s College London, London SE1 1UL, UK

## Abstract

Cell-matrix adhesion is essential for building animals, promoting tissue cohesion, and enabling cells to migrate and resist mechanical force. Talin is an intracellular protein that is critical for linking integrin extracellular-matrix receptors to the actin cytoskeleton. A key question raised by structure-function studies is whether talin, which is critical for all integrin-mediated adhesion, acts in the same way in every context. We show that distinct combinations of talin domains are required for each of three different integrin functions during *Drosophila* development. The partial function of some mutant talins requires vinculin, indicating that recruitment of vinculin allows talin to duplicate its own activities. The different requirements are best explained by alternative mechanisms of talin function, with talin using one or both of its integrin-binding sites. We confirmed these alternatives by showing that the proximity between the second integrin-binding site and integrins differs, suggesting that talin adopts different orientations relative to integrins. Finally, we show that vinculin and actomyosin activity help change talin’s orientation. These findings demonstrate that the mechanism of talin function differs in each developmental context examined. The different arrangements of the talin molecule relative to integrins suggest that talin is able to sense different force vectors, either parallel or perpendicular to the membrane. This provides a paradigm for proteins whose apparent uniform function is in fact achieved by a variety of distinct mechanisms involving different molecular architectures.

## Highlights

• Integrin function requires distinct sets of talin domains in three different tissues
• Vinculin helps talin retain function when domains are removed
• Talin IBS2 is separated from integrins in muscle but not wing adhesion sites
• Vinculin and actomyosin contribute to separating IBS2 from integrins

## Graphical Abstract

**Figure:**
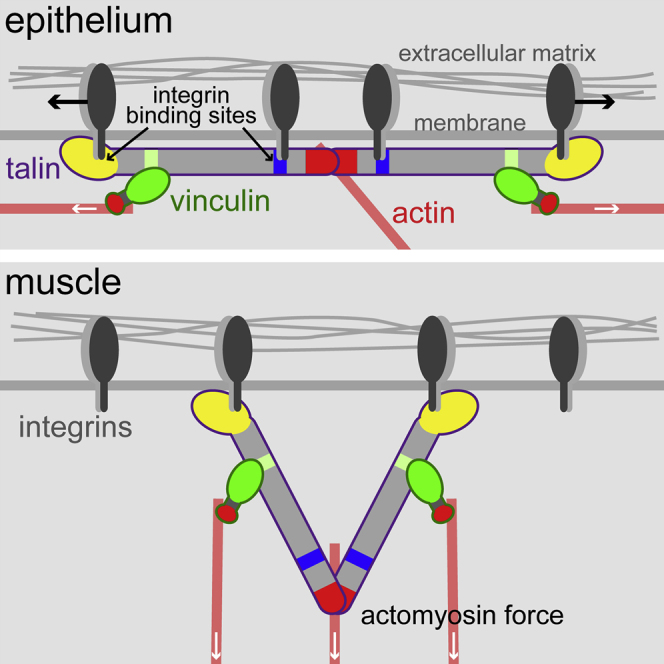

## Teaser

Klapholz et al. show that talin domains have variable importance in different developmental events in *Drosophila*, suggesting distinct mechanisms of action, with altered configurations of talin, vinculin, and integrins. These were validated by advanced imaging in vivo. Such diverse configurations may allow talin to sense a variety of forces.

## Introduction

**Figure 1:**
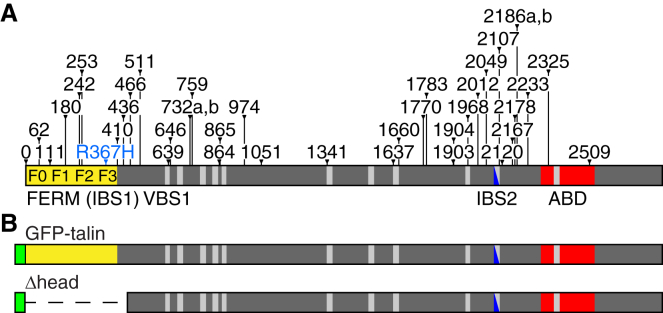
New Talin Mutants (A) The diagram shows the domains of talin: F0–F3 of the FERM domain (yellow), vinculin-binding sites (light gray), integrin-binding site 2 (dark blue), and actin-binding domain (red). IBS2 is also a VBS. Mutations are indicated above talin: 38 cause a truncation (black), and one causes a substitution (R367H; blue). The truncations are named according to their last in-frame talin residue (see Table S1). (B) GFP-talin and Δhead transgenes. The position of GFP (green) and deletion (dashed line) are indicated.

In multicellular organisms, cells adhere to extracellular matrices (ECMs) to migrate and resist mechanical force. ECM adhesion is generally mediated by integrins, transmembrane receptors connecting the ECM to the actin cytoskeleton via multiple intracellular linker proteins [1, 2]. One intracellular adaptor, talin, is particularly critical for this connection, being uniquely essential for all integrin adhesive functions within developing organisms [3, 4]. Talin is a large multidomain molecule that makes numerous protein interactions and has at least two separable functions: modulating integrin affinity and linking integrins to actin [5]. The N-terminal “head” domain is a modified FERM domain (band4.1, Ezrin, Radixin, Moesin) with four subdomains, F0–F3 [6] (Figure 1A). An F2-F3 fragment binds the integrin β subunit cytoplasmic tail, with integrin-binding site 1 (IBS1) within F3, and is necessary and sufficient for “inside-out” integrin activation, increasing ECM binding [7]. The head also contains membrane-binding sites in F1 and F2, and binds actin and other proteins [5]. The rest of talin, the C-terminal “rod,” is composed of α-helical bundles, which include binding sites for vinculin, integrin (IBS2/α helix 50 [8]), and actin [9]. The vinculin-binding sites (VBSs) are buried within the helical bundles but are exposed by force across talin, contributing to the force dependency of vinculin recruitment [10, 11].

These findings led to a model where (1) talin binds integrins via the head domain, activating integrins; (2) the C-terminal actin-binding domain (ABD; distinct from two additional actin-binding regions, in the head and central rod) binds to actin; and (3) force from actin polymerization or myosin contraction stretches talin, exposing VBSs that recruit vinculin, providing additional links to actin. In addition to vinculin and actin, talin recruits other integrin-associated proteins [12], providing a scaffold for protein complex assembly.

This model agrees with superresolution microscopy showing talin oriented perpendicular to the plasma membrane, with the head bound to integrin and the ABD to actin [13, 14]. However, it does not explain how IBS1-mutant talin is still recruited to adhesions [15], how the isolated C terminus of the talin rod can mediate cell proliferation [16], or why in *Drosophila*, IBS2 is required for more integrin-mediated processes than IBS1 [17]. Moreover, site-directed talin mutants retain partial activity, which varies with the developmental event examined [15, 17, 18]. Thus, it is likely that talin function is more complex: different domains of talin may operate independently; different tissues or developmental stages may express “redundant” proteins that substitute for distinct talin subfunctions; or talin may function by more than one molecular mechanism, with different domains being more or less important for each mechanism. Our findings show that indeed, within the different cells of an organism, the way that talin assists integrins to mediate adhesion varies dramatically.

## Results

### New Talin Mutant Alleles

To identify key residues required for talin function, we exploited *Drosophila* genetics to generate cells homozygous for randomly generated mutations just in the wing and selected mutants impairing integrin adhesion. From 50,000 mutants screened, 39 talin mutants were isolated. To our surprise only two changed a single residue (Figure 1A; Table S1), and one of these changed the initiating methionine, preventing translation (talin^0^). The other (talin^R367H^) altered a key residue in IBS1, changing R367 to histidine (R358 in human talin [19, 20]), similar to the talin^R367A^ mutant we generated previously to impair integrin activation [15]. The other 37 mutations were truncations caused by stop codons or frameshifts, providing an invaluable deletion series from the C terminus, which enabled the mapping of key activities, as described below. For comparison, 19 of 38 of the other mutants from the screen were single-residue changes (unpublished observations). This suggests that there are few single residues that are critical for talin function or structure. To complement this series of C-terminal deletions, we generated a site-directed *GFP-talinΔhead* (*Δhead*) allele, expressed from the talin promoter and tagged with GFP, as well as the wild-type control construct *GFP-talin* (Figure 1B), and combined them with a null allele in the endogenous gene. *GFP-talin* fully rescued the null allele, whereas *Δhead* was lethal with the phenotypes described below.

None of the mutant talins caused dominant effects; all are recessive alleles. We used them to assay the function of different regions of talin in three distinct integrin-mediated developmental processes: (1) muscle attachment in the embryo; (2) epidermal morphogenesis during early embryogenesis; and (3) adhesion between the two epithelial cell layers of the adult wing. Surprisingly, each process required different talin domains.

### Vinculin Partially Substitutes for the Loss of Talin’s Direct Actin Binding in Muscles

**Figure 2:**
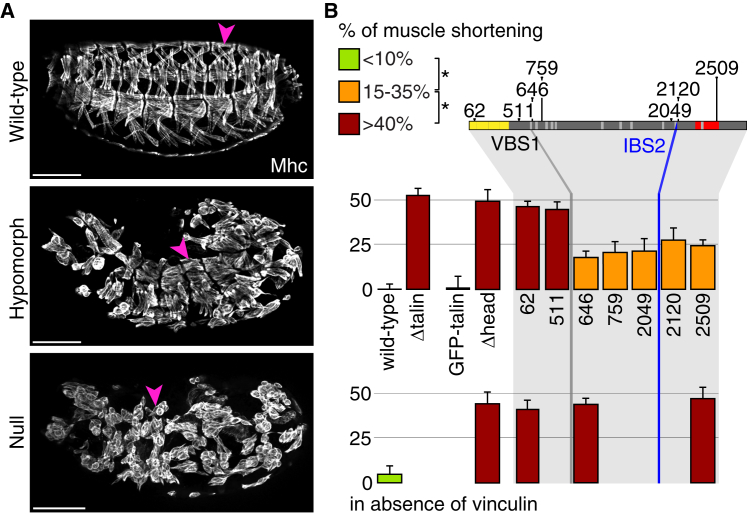
Talin Head, but Not IBS2, Is Essential for Integrin Function at Muscle Attachment Sites (A) Weak versus strong muscle attachment defects in talin mutant embryos. Muscle myosin heavy chain (Mhc) staining of embryonic muscles exhibiting no defect (wild-type; top), mild detachments (hypomorphic phenotype; center), or complete detachment (null phenotype; bottom). The scale bars represent 100 μm. (B) The average shortening of five dorsal muscles (pink arrowheads in A) was quantified per embryo homozygous for the indicated mutants and plotted as the reduction in muscle length relative to wild-type in the presence (top histogram) or absence (bottom histogram) of vinculin. Bar colors show three statistically distinct categories (^∗^p < 0.01; green bars are not significantly different from wild-type). At least five embryos were measured per genotype. Error bars are SD. Genotypes not analyzed do not have a bar.

The most prominent embryonic integrin-adhesion structures are the muscle attachment sites (MASs); without integrin or talin, the muscles fully detach. Many talin mutants retained some muscle attachment (Figure 2A; Figure S1), quantified by measuring shortening of dorsal muscles (Figure 2B). Three phenotypic classes were statistically distinct (p < 0.01): null, partial loss of function, and wild-type, shown by bar color. Deletion of the head completely inactivated talin; Δhead protein levels were normal and the remaining rod fragment was recruited (Figures S1 and S2) but had no detectable function. This is much stronger than the point mutant in IBS1 [15], consistent with the head having other activities in addition to binding and activating integrin, such as membrane binding [6, 21, 22]. The most C-terminal truncated protein, talin^2509^, which lacks half of the ABD dimerization helix, still had some function in muscle adhesion (Figure 2B). Deletion of the whole ABD in talin^2120^ did not impair talin function further, consistent with the dimerization helix being essential for actin binding [23, 24], and possibly only necessary for this function, because a point mutant that inactivates actin binding but not dimerization is equivalent to one that impairs both [18]. The deletion that also removes IBS2 retained the same level of partial activity (compare 2049 and 2120), even though a site-directed IBS2 mutant caused muscle detachment [17] (discussed below). Further deletion from the C terminus revealed an abrupt transition from partial activity to no activity when the last VBS was deleted, going from talin^646^ to talin^511^. This transition did not correlate with protein levels, because talin^511^ was expressed similarly to talin^759^ (Figure S2) but caused stronger detachment (Figure 2B). Thus, C-terminal deletions revealed two steps: talins lacking the ABD retained partial function, which was lost only when the last VBS was deleted. This suggested that vinculin binding compensates for ABD deletion, so we tested vinculin’s contribution.

To avoid any concern of partial vinculin activity in the existing *Vinculin* mutant [25], we generated a deletion removing all of the *Vinculin* coding sequence, *ΔVinc*, which is viable and does not cause any visible phenotype in the adult. Removal of vinculin from talin mutants that lacked the ABD but contained one or more VBSs caused the loss of the residual talin function (Figure 2B). This was not due to a nonspecific additive effect, as removing vinculin did not enhance every talin mutant with partial activity (see below). We therefore conclude that in the muscles, vinculin is partially compensating for the absence of ABD, possibly by using its own ABD.

In summary, the muscle phenotype of the new talin mutants fully fits the model of talin function in focal adhesions outlined in the Introduction, as the head is critical and there is some overlap in the function of the ABD and bound vinculin. However, this is not the case for other developmental processes.

### In Epidermal Morphogenesis, Vinculin Can Substitute for Talin Head Function

**Figure 3:**
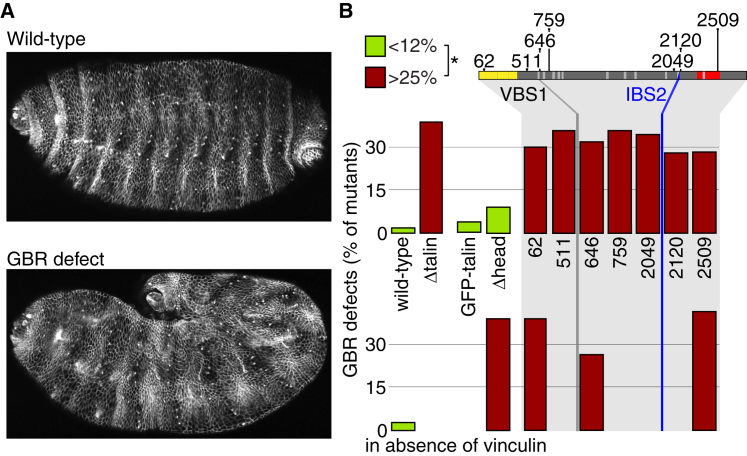
In Germband Retraction, Talin Head and Vinculin Are Redundant, whereas ABD Is Essential (A) Embryos exhibiting no defect (wild-type; top) or a GBR defect (bottom), stained for Fasciclin 3 in lateral epidermal membranes. (B) The percentage of GBR defects was quantified in ≥50 embryos/mutant. Bar colors show two statistically distinct categories (^∗^p < 0.01; green bars are not significantly different from wild-type). Genotypes not analyzed do not have a bar.

We next investigated the contribution of talin domains to the morphogenetic process of germband retraction (GBR) of the embryo, which reverses the elongation of the germband that occurred during gastrulation (Figure 3A). Quantifying embryos with GBR defects showed that the alleles caused one of two effects, either indistinguishable from the null talin allele or wild-type (Figure 3B). In embryos with the talin gene completely deleted (Δtalin), 38% failed to undergo GBR, showing that talin makes an important contribution to this process, but there must be a compensating factor that allows many embryos lacking talin to undergo GBR. In contrast to the muscle, loss of the head (Δhead) had no effect on talin’s contribution to GBR (Figure 3B), whereas the most C-terminal truncated protein, talin^2509^, had no GBR activity. These findings were consistent with previous work showing that specific disruption of actin binding caused a null GBR defect [18], but contrasted with the null GBR defect seen in embryos expressing headless-talinGFP, a construct similar to our Δhead [26]. The difference could be caused by the GFP tag inserted at the C terminus of headless-talinGFP, which may partially impair actin binding [18, 27].

As expected, the failure of talin^2509^ to mediate GBR did not get worse by removing vinculin, but surprisingly Δhead lost all its activity (Figure 3B). Vinculin is not known to bind integrins, suggesting that vinculin is substituting for another function of talin’s head. Both talin head and vinculin bind actin and the membrane, suggesting that one of these activities is essential for GBR.

### IBS2/α Helix 50 Is Important for Talin Function in the Wing

**Figure 4:**
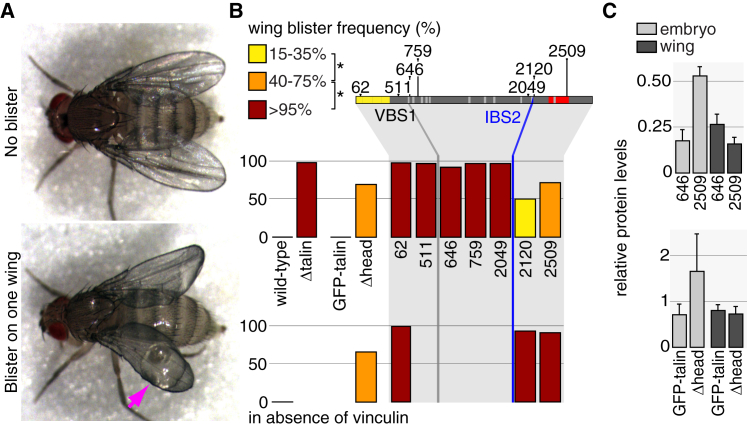
Both Talin Head and IBS2 Are Used for Wing Adhesion (A) Representative pictures of newly hatched flies with normal wings (top) or one wing with a blister (bottom; pink arrow). (B) Percentage of blistered wings in flies with homozygous mutant clones for the mutants indicated (for all mutants, see Figure S3), from ≥100 flies/mutant. Bar colors show four statistically distinct categories (^∗^p < 0.01). Flat bars (horizontal lines) indicate no defect. Genotypes not analyzed do not have a bar. (C) Phenotypic differences between muscle and wing are not explained by differences in protein levels, determined by western blotting of talin^646^ and talin^2509^ (top histogram) and talin site-directed mutants (bottom histogram) in embryos (light gray) or pupal wings (dark gray) heterozygous for the talin mutation. The protein levels were normalized to wild-type talin in each sample. SD is shown from two independent experiments.

We next examined talin mutant function in wing adhesion. Because talin is required for viability, these experiments were performed by inducing homozygous mutant cells within the developing wing and assaying the wing blister phenotype (Figure 4A). Quantitation of all talin mutations revealed four statistically distinct phenotypic classes, indicated by three bar colors and the absence of a bar, and showed that many talin truncations retained some adhesive function (Figure 4B; Figure S3). Intriguingly, the requirement for particular talin domains was different from muscle or GBR (Figure S4).

In contrast to both the roles for talin head in muscle and GBR, Δhead had partial activity in the wing (Figure 4B). The C-terminal deletions that just impair ABD had partial activity, similar to Δhead. Of interest, talin^2120^ had more activity than truncations up to talin^2167^ (Figure 4B; Figure S3), suggesting an inhibitory domain between 2120 and 2167. Uniquely in this tissue, we observed the abrupt transition from partial to null activity at the transition from talin^2120^ to talin^2049^ (Figure 4B). Notably, the 71-residue region between these deletion endpoints contains α helix 50, which has residues critical for IBS2 function [8, 28] and is a VBS [29]. This suggests that binding of integrin, vinculin, or another molecule is critical, although the existence of many other VBSs in this truncation argues against it being vinculin. These results suggest that both IBSs contribute to talin function in the wing. We then tested whether they needed to be in the same molecule by measuring whether the partial blister phenotype of Δhead could be ameliorated by combining it with a truncation producing talin head, talin^646^, but it was not (Figure S4). This demonstrates that for full function the head and rod must be in the same molecule. The remaining function of truncations lacking the ABD required vinculin, similar to muscle, but in contrast to GBR the remaining function of Δhead did not require vinculin (Figure 4B). This finding was also important because it showed that removing vinculin does not enhance every talin mutant that retains partial activity.

To summarize, each developmental process requires a unique set of talin regions. Three key mutants reveal these differences: (1) Δhead completely inactivated function in muscle, was fully functional for GBR as long as vinculin was present, and had partial function in the wing, regardless of vinculin’s presence; (2) the most C-terminal truncated protein, talin^2509^, which impairs actin binding, had partial vinculin-dependent function in muscle and wing and no function in GBR; and (3) the mutant talin lacking the ABD and IBS2/α helix 50, talin^2049^, retained the partial activity of ABD deletions in muscle, had the same null defect as ABD deletions in GBR, and eliminated the partial activity in the wing. These differences suggested that the mechanism of talin function in each process could be different. We therefore considered alternative models of talin function to explain these differences and focused on the differences between muscle and wing, because they both involve clear integrin-containing adhesive structures that mediate strong adhesion between tissue layers.

We first checked that these differences in activity of mutant talins are not caused by altered protein stability at different developmental stages. Phenotypic differences in muscle versus wing for Δhead (null versus partial activity) and talin^646^ (partial activity versus null) were not explained by reduced talin levels in the tissue with the stronger phenotype (Figure 4C).

In the wing, both the residual activity of Δhead, which lacks IBS1, and the importance of IBS2 support a key role for IBS2 binding to integrin. One way to explain the results is if in muscles a single talin molecule lacking its ABD and IBS2 can link an integrin to actin with IBS1 and vinculin (providing reduced but significant function); in contrast, this does not work in the wing, where instead each talin molecule must bind two integrins. This latter point arises because we note that every talin mutant that retained partial activity in the wing can make a talin dimer/monomer with two IBSs: Δhead still has the dimerization helix and so can make a homodimer with two IBS2s, whereas deletion of ABD results in a monomer containing IBS1 and IBS2. It also fits with our finding that for full function, both IBSs have to be in the same molecule. We therefore tested whether the proximity between integrin and IBS2 varied in the two tissues by measuring fluorescence resonance energy transfer (FRET) within the whole animal.

### FRET-Fluorescence Lifetime Imaging Reveals Close Proximity between IBS2 and Integrin in Wings but Not Muscles

We quantified FRET by fluorescence lifetime imaging (FLIM), which measures the reduction in lifetime of the donor fluorescence when FRET occurs between two fluorescent molecules less than 10 nm apart [30]. Fortuitously, a gene trap insertion was isolated that permits the insertion of mCherry in-frame into talin, 18 amino acids C-terminal to IBS2/α helix 50 (talinIBS2-mCherry [31]). In addition, we generated an integrin βPS subunit tagged with GFP at the C terminus (βPS-GFP) by homologous recombination and genomic rescue constructs encoding vinculin tagged with GFP or red fluorescent protein (RFP) at the C terminus. The fluorescent tags did not impair function, as the insertions into the integrin and talin genes were homozygous viable and fertile with no visible defect, and the tagged vinculins tightly colocalized with integrins.

**Figure 5:**
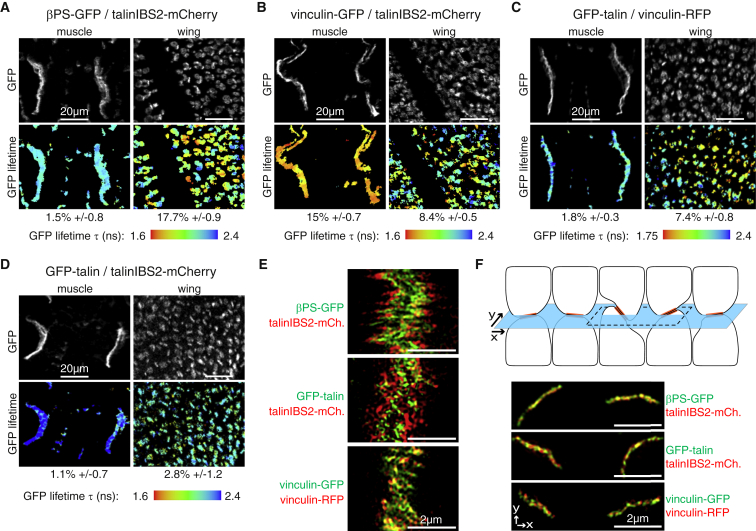
Talin IBS2 Is in Proximity to Integrins in Wing but Not in Muscle (A–D) FRET analysis to determine the proximity between βPS-GFP and talinIBS2-mCherry (A), vinculin-GFP and talinIBS2-mCherry (B), GFP-talin and vinculin-RFP (C), or GFP-talin and talinIBS2-mCherry (D) at integrin adhesion sites in muscle (left panels) and wing (right panels). Donor GFP (gray in top panels) and GFP lifetime heat maps (lower panels, with scale in ns) are shown. Increasing FRET shortens GFP lifetime; FRET efficiencies are indicated by the standard errors (n > 10) below the panels. (E) 3D-SIM shows that βPS-GFP (top) and GFP-talin (center) are separated from talinIBS2-mCherry at MASs, in contrast to vinculin-GFP and vinculin-RFP (control for chromatic aberration; bottom). (F) Diagram of integrin adhesions (orange) between the two epithelial cell layers of the wing. The x-y focal plane of the images is shown with the light blue horizontal plane. Some adhesions are tilted sufficiently to provide a transverse section (dashed black rectangle). Pictures: 3D-SIM x-y sections through wing adhesions show colocalization of βPS-GFP and talinIBS2-mCherry (top), GFP-talin and talinIBS2-mCherry (center), and vinculin-GFP and vinculin-RFP (bottom).

The βPS-GFP/talinIBS2-mCherry pair did not show FRET in muscles, but showed substantial FRET in wing adhesions (Figure 5A). Thus, talin’s IBS2 is in closer proximity to integrin in wing versus muscle, supporting the increase in phenotype we observed when IBS2 was deleted in wing but not muscle. The degree of proximity varied between different wing adhesions, suggesting a dynamic interaction. The pattern varied from wing to wing, and this variability was found in live wings as well as at earlier and later pupal stages (data not shown).

We then examined whether vinculin was in close proximity to talin head or IBS2 by analyzing two FRET pairs: vinculin-GFP/talinIBS2-mCherry and GFP-talin/vinculin-RFP. Vinculin’s C terminus was in close proximity to IBS2 in both tissues (Figure 5B), demonstrating that we can detect FRET at muscle adhesions, and therefore there is no technical reason for not detecting FRET there between integrin and IBS2. Vinculin’s C terminus was also in close proximity to talin head, but only in the wing (Figure 5C), consistent with distinct molecular architectures in the two tissues. The FRET of these pairs showed a similar level of variability in the wing as βPS-GFP/talinIBS2-mCherry, suggesting integrin adhesions are generally more dynamic in wing versus muscle. Finally, the GFP-talin/talinIBS2-mCherry pair did not show FRET in either wing or muscle (Figure 5D), indicating that talin head is not close to IBS2, and confirming that the FRET we did observe in the wing is not due to any nonspecific crowding effect.

The lack of IBS2 proximity to integrin in muscles does not explain the previous result that an IBS2 point mutant has a strong muscle phenotype [17]. To resolve this contradiction, we hypothesized that, in the muscle, talin initially binds to integrin via IBS2, and then actin binding via the ABD and vinculin pulls the talin C terminus away from the membrane (see Discussion). This prompted a number of new experiments to determine the extent of the separation between IBS2 and integrins, and test whether actomyosin activity and vinculin are involved in this separation.

### Superresolution Microscopy Shows that IBS2 Is Separated from Integrins in the Muscles but Not the Wings

We used superresolution 3D structured illumination microscopy (3D-SIM [32]) and observed at MASs a clear separation between βPS-GFP and talinIBS2-mCherry (in 26 of 29 MASs analyzed) and between the two ends of talin, GFP-talin/talinIBS2-mCherry (in 8 of 9 MASs). In contrast, no separation was detected (0 of 27 MASs) between a combination of vinculins C-terminally tagged with GFP or RFP (Figure 5E). 3D-SIM has a resolution of 120 nm, consistent with separation of talin ends by >250 nm in mammalian cells [33], which is stretched relative to the ∼60-nm length by electron microscopy [34]. This indicates that talin is stretched perpendicular to muscle ends, resulting in the separation of IBS2 from integrins. In contrast, in the wing, we never observed a separation between βPS-GFP and talinIBS2-mCherry (n = 15 wings) or GFP-talin and talinIBS2-mCherry (n = 5 wings) (Figure 5F). This fits with the fact that IBS2 contributes to function in the wing and suggests that talin head is localized close to integrins at the membrane. Thus, these observations show that the differences in the regions of talin that are crucial in the two tissues are reflected by a difference in the configuration of talin, suggesting that talin is oriented perpendicular to the membrane in muscles and parallel in wings.

### Myosin and Vinculin Separate IBS2 and Integrins in Muscle

**Figure 6:**
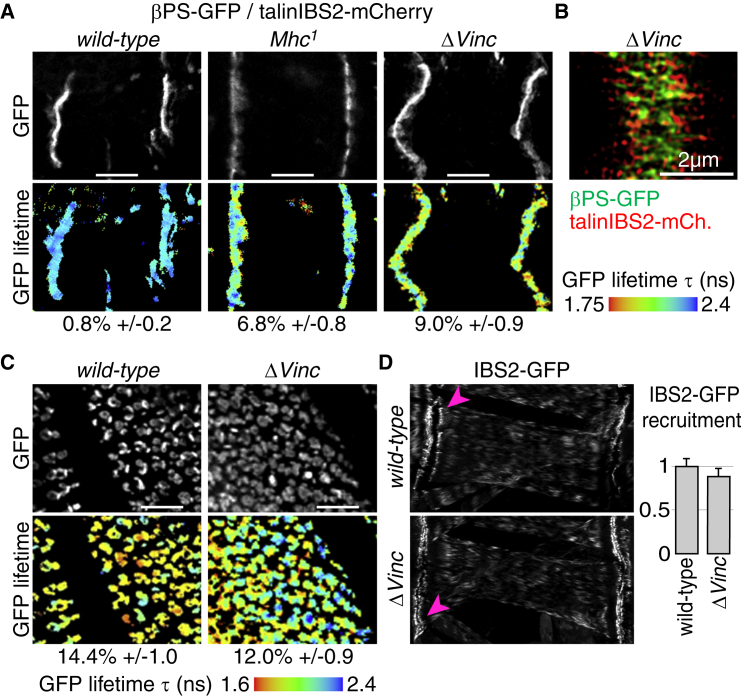
Muscle Myosin Heavy Chain and Vinculin Are Required to Separate IBS2 from Integrins at Muscle Attachments (A) FRET analysis at MASs shows that talinIBS2-mCherry and βPS-GFP are in closer proximity in the absence of muscle myosin (*Mhc*; middle panels) or vinculin (right panels) compared to *wild-type* (left panels). Donor GFP is shown in gray on top and GFP lifetime heat maps below. Increasing FRET shortens the lifetime; FRET efficiencies are indicated by the standard errors (n > 15) below the panels. The scale bars represent 20 μm. (B) 3D-SIM shows that βPS-GFP and talinIBS2-mCherry are separated at MASs in the absence of vinculin. (C) FRET analysis at wing adhesions shows that the proximity between talinIBS2-mCherry and βPS-GFP is not affected in the absence of vinculin. The scale bars represent 20 μm. (D) Pictures: muscle-specific overexpression of IBS2-GFP at MASs (pink arrowheads) in the presence (*wild-type*; top) or absence (*ΔVinc*; bottom) of vinculin. Histogram: quantitation of IBS2-GFP levels at MASs, normalized to wild-type levels. Error bars are SDs (n > 25).

The separation between integrins and IBS2 at MASs could result from forces exerted on the rod of talin, pulling it away from the membrane. When we disrupted the contractile apparatus of muscles, by removing muscle myosin [35], we could now detect FRET between βPS-GFP and talinIBS2-mCherry (Figure 6A), showing that they have moved closer together. We hypothesized that actomyosin’s contribution could be mediated directly via talin’s ABD and/or indirectly via vinculin’s ABD. Supporting the latter, removing vinculin also resulted in integrin and IBS2 coming together (Figure 6A), comparable to the FRET observed in muscle myosin mutants. It appears that only a fraction of talin becomes oriented with IBS2 close to integrin, because βPS-GFP and talinIBS2-mCherry remained separated at MASs in vinculin mutants when visualized with superresolution microscopy (in 17 of 24 MASs; compare Figures 5E and 6B). It proved not possible to do 3D-SIM in muscle myosin mutants, because the βPS-GFP/talinIBS2-mCherry fluorescence intensity was too low.

An alternative way that loss of vinculin could increase the fraction of talins with IBS2 in close proximity to integrin is if vinculin competes with integrins to bind α helix 50/IBS2, as this helix is also a VBS [29]. To test whether vinculin competes with integrins for IBS2, we determined whether removing vinculin increased βPS-GFP/talinIBS2-mCherry FRET in the wing (Figure 6C) or increased IBS2-GFP [15] recruitment to MASs (Figure 6D), and found that it did not. The lack of competition may suggest that the vinculin-GFP/talinIBS2-mCherry FRET signal derives from the close proximity between vinculin-GFP bound to another VBS and the mCherry inserted near IBS2. Altogether, our data support a mechanism by which actomyosin contractions and vinculin separate IBS2 from integrins in muscle, most likely by exerting force on the C terminus of talin that pulls it away from integrins.

## Discussion

We have presented key findings that change our view of talin function: (1) talin is needed for every integrin adhesion event in fly development, each with variable dependence on individual talin interaction sites; (2) the IBS2 of talin is separated from integrins in muscle but not in wing, and this partly requires myosin activity and vinculin; and (3) even though the absence of vinculin is tolerated, vinculin is required for certain mutant talins to retain their residual function.

Vinculin’s maintenance through evolution in *Drosophila* was at odds with the lack of a mutant phenotype [25], especially as vinculin mutants are lethal in other organisms [36, 37]. However, vinculin mutants have recently been observed to cause mild muscle detachment in late-stage fly larvae [38], and here we show that vinculin is required for the partial activity of talin mutants. Thus, vinculin supports normal functions of talin by adding additional actin/membrane-binding sites. Activated vinculin increases focal adhesion size, slows talin turnover, and maintains stretched talin in an unfolded conformation [39–41], and so vinculin may also increase the stability of mutant talins at adhesion sites. The ability of vinculin to aid mutant talin function is somewhat paradoxical if stretch between head and ABD is required to expose VBSs [10, 11]: how therefore do talins that lack the C-terminal ABD recruit vinculin? Possible explanations include: (1) some VBSs are exposed in unstretched talin; (2) other interactions stretch and expose VBSs; (3) truncation exposes VBSs; and (4) activation of vinculin drives binding to truncated talins, because artificially activated vinculin can recruit talin [39].

Our finding that the C terminus of vinculin was in close enough proximity to talin to show FRET was surprising, because the talin-binding domain of vinculin is at its N terminus and therefore the actin-binding C terminus would be expected to extend away from talin. In all our other ongoing experiments, we only get FLIM if the tag is adjacent to the interaction site (our unpublished observations). The close proximity therefore suggests that vinculin becomes aligned with talin. In muscle and wing, this alignment would be in the same direction, with vinculin binding a VBS N-terminal to IBS2, resulting in vinculin’s C terminus in close proximity to the mCherry inserted C-terminal to IBS2. This is consistent with actin-mediated forces pulling the C-terminal ABDs of talin and vinculin away from integrins and talin head, respectively. The FRET indicates that some vinculin is pulled in the opposite direction in wings but not muscles, bringing vinculin’s C terminus near talin’s N terminus. This difference fits talin’s parallel orientation in the wing, where the cortical actin meshwork could pull vinculin in a variety of directions. It is also possible that talin’s head and vinculin’s C terminus are brought into proximity by membrane binding.

Our results provide additional support for binding of IBS2 to integrins [30, 42], consistent with results showing that mutating IBS2 and the IBS2-binding site on the βPS integrin subunit cytoplasmic domain have similar phenotypes [17]. We show that continued interaction between IBS2 and integrins is context dependent, with lack of IBS2 proximity to integrins at MASs, as in focal adhesions [13, 14], and retention of proximity in the wing. Our finding that IBS2 was not required in the embryo for the residual function of talin lacking ABD, or talin/PINCH maintenance in this mutant (Figure S1 and not shown), seems inconsistent with the defects caused by an IBS2 site-directed mutation, including muscle detachment and separation of talin and PINCH from integrins [17]. Furthermore, we need to explain how IBS2 can be required for talin to remain bound to integrins [17] but not remain in close proximity. One explanation is to hypothesize that IBS2-integrin binding strengthens the interaction of talin’s head with another integrin or the plasma membrane, so that it can resist the pulling forces on ABD and vinculin that separate IBS2 away from integrins. When IBS2 is mutated the interaction between talin head and integrins/membrane is weakened, such that the full-length protein is pulled off, but a protein lacking ABD remains attached sufficiently to provide some function. This suggests that IBS2 should be in close proximity to integrins during early stages of adhesion formation in muscles, but we were unable to detect any FRET (unpublished observations). It could therefore be a transient interaction or IBS2 may bind another protein in muscles.

**Figure 7:**
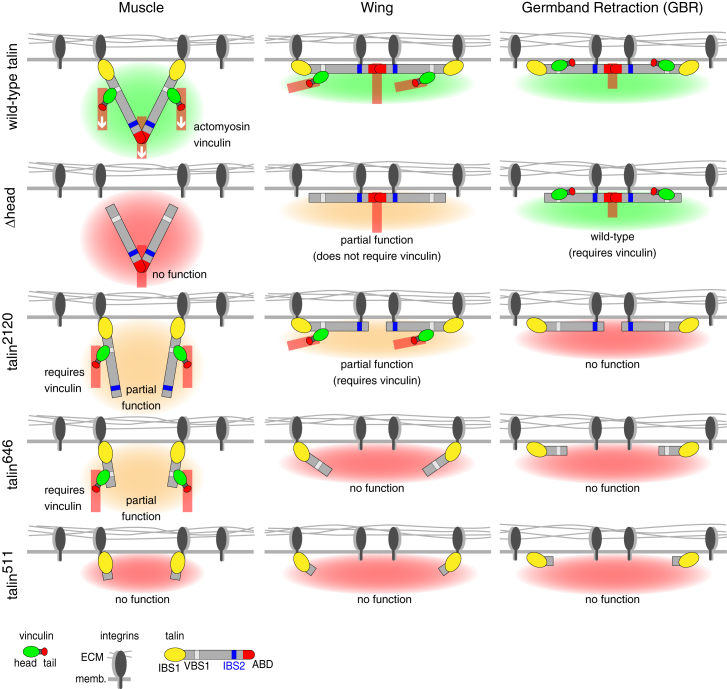
Models for Three Mechanisms of Talin Action The top diagrams show models for the way talin functions in muscle and wing and during germband retraction. At muscle attachment sites (left), talin dimers are bound to integrins or membrane with their heads and to actin directly with the C-terminal ABD and indirectly with vinculin. Actomyosin activity and vinculin are important to separate IBS2 and integrins, likely by exerting force on the rod of talin that pulls it toward the inside of the cell (white arrows). In the wing (middle), talin is oriented parallel to the membrane, with each talin dimer using all IBSs. This talin dimer binds actin, directly with the C-terminal ABD and indirectly with vinculin. During germband retraction (right), talin dimers are bound to actin or membrane directly with the head and indirectly with vinculin (only membrane binding is shown), to integrins with IBS2s, and to actin with the C-terminal ABD. In wing and germband retraction (see below), we suggest IBS2 binds integrins but could interact with other functional binding partners. The logic that generated these models from the mutant phenotypes is demonstrated by depicting the phenotypic effect of the mutations in each model. In muscle, talin head is essential to bind integrins and membrane, as its absence (Δhead) resulted in a null phenotype (red background). The absence of ABD (talin^2120^) resulted in a hypomorphic phenotype (orange background), and this remaining function requires vinculin but it is not clear how vinculin is recruited. The additional loss of IBS2 (talin^646^) did not enhance the phenotype, but talin caused a null phenotype when all VBSs were deleted (talin^511^). In the wing, talin head is important but integrins can still be linked together through dimerized IBS2s. The absence of ABD resulted in a hypomorphic phenotype, and this remaining function requires vinculin. The additional loss of IBS2 resulted in a null phenotype, as integrins cannot be linked together by talin. During germband retraction, the head was not required (green background) but vinculin was essential in this context. It is not clear how vinculin is recruited to Δhead. All truncations deleting ABD activity resulted in a null phenotype.

We propose three distinct models for the mechanisms adopted by talin to mediate integrin adhesion, and these explain all our findings (Figure 7). (1) In muscle, talin appears to work as presented in the Introduction, with talin dimers bound to integrins or membrane with their heads and to actin directly with the C-terminal ABD and indirectly with vinculin. Actomyosin activity and vinculin likely exert force on the rod of talin, each separating a fraction of the IBS2s from integrins. (2) In the wing, talin is oriented parallel to the membrane, with each talin dimer binding four integrins using all IBSs. Alternatively, talin heads are bound to the membrane or cortical actin, and the IBS2s are bound to two integrins. Actin is bound directly with the C-terminal ABD and indirectly with vinculin. (3) During GBR, we suggest that talin dimers are bound to cortical actin or membrane directly with the head and indirectly with vinculin. Because IBS2 is critical for GBR [17], we further suggest that talin dimers bind to integrins with IBS2s and to actin with the C-terminal ABD. In these models, we have opted for the simplest explanation where IBS2 binds directly to integrins, but we have not ruled out that there are intermediate adaptor proteins.

In the wing, the proximity between IBS2 and integrins could result from insufficient actomyosin activity perpendicular to the membrane, but such a “passive” mechanism could not explain why IBS2 was critical in some tissues. The requirement for both talin head and IBS2 in the wing and during GBR suggests new parallel orientations of talin that could sense stretching forces within the adhesion plane, similar to EPLIN at cell-cell adhesions [43]. In the wing, stretch would occur between integrins, and between integrin and membrane or actin in GBR. It is also possible that talin senses stretch between the membrane and cortical actin, as organisms lacking integrins have talin [44]. The different orientations will also impact on integrin density and integrin:talin stoichiometry. In the wing, the distance between integrins can be fixed by talin, whereas in the muscle, integrin density would vary, depending on the flexibility of the talin dimer. It will be of interest to find whether parallel orientation of talin is found in epithelia of other organisms.

Finally, our results emphasize that when mutant versions of a protein are found to work better in some cell types than others, this may be indicating different mechanisms of action, a possibility that could resolve apparently contradictory findings.

## Experimental Procedures

### *Drosophila* Genetics

Details on the generation of new *rhea* (*talin*) and *Vinculin* alleles can be found in Supplemental Experimental Procedures.

For wing blister quantification, mitotic clones were generated in the wings of heterozygous flies by crossing *rhea* mutant males to *w; P{w[+], Gal4}Vg[BE] P{w[+], UAS::FLP}; P{FRT}2A* (with the *white+* excised from *P{FRT2Aw[hs]}*) females. Embryonic phenotype quantification was performed on mutant embryos lacking both maternal and zygotic wild-type talin and/or vinculin, as they were obtained from germline clones generated in heterozygous mutant females by crossing *rhea* mutant females (with wild-type *Vinculin* or *ΔVinc*) to *P{hs::FLP}1, y[1] w[118]; P{ovoD1-18}3L P{FRTw[hs]}2A* (for genotypes with wild-type *Vinculin*) or *ΔVinc w[-]; P{hs::FLP}38/CyO; P{ovoD1-18}3L P{FRTw[hs]}2A* (for genotypes with *ΔVinc*) males. Heat shocks were performed two times for 1 hr and 15 min each at 37°C at L1 and L2 larval stages. TalinIBS2-mCherry [31] was kindly provided by H.J. Bellen. The *myosin heavy chain* mutant used was *Mhc[1]* [45], kindly provided by S.I. Bernstein. IBS2-GFP recruitment to muscle attachment sites was performed with *UAS::IBS2-GFP* [15] expressed in muscles with *P{Gal4-Mef2.R}3* (Bloomington *Drosophila* Stock Center).

### Molecular Cloning

Details on the generation of genes expressing fluorescently tagged talin, vinculin, and βPS integrin subunit are in Supplemental Experimental Procedures.

### Stainings, Confocal Microscopy, and Image Analysis

Immunostainings were carried out according to standard procedures, as fully described in Supplemental Experimental Procedures.

Primary antibodies were rabbit anti-talin N terminus [46] (1:75), rabbit anti-GFP (1:500; Ab290; Abcam), mouse anti-muscle myosin [47] (1:100; FMM5), and rat anti-αPS2 [48] (1:15; 5D6). Samples were scanned with an Olympus FV1000 confocal microscope using a 20×/0.75 NA objective with 1.2× zoom for whole-embryo pictures or a 60×/1.35 NA objective with 2× zoom for muscle attachments. The images were processed with ImageJ (NIH) and Adobe Photoshop. The lengths of embryonic dorsal muscles were measured with ImageJ from raw z stacks. The average muscle shortening and standard deviation for each genotype were obtained from five embryos, in each of which five dorsal muscles were measured to calculate a mean length per embryo. Each dorsal muscle length was normalized by the mean length of the embryo and compared to wild-type to calculate the percentage of shortening for each genotype. Germband retraction defects were scored by counting embryos (n > 50) stained with anti-talin N terminus, which exhibits a background staining outlining the epidermis. The quantitation of IBS2-GFP recruitment to MAS was performed on dorsal MASs of 13–15 live 0- to 1-hr-old larvae. Two five-frame stacks per larvae were imaged (n = 25–26) and analyzed with MATLAB (MathWorks).

### Statistical Tests

Statistical differences in muscle shortening (three significantly different classes) were determined by Student tests (p < 0.01) using Excel (Microsoft). Statistical differences in the frequencies of wing blisters (four classes) or GBR defects (two classes) were determined by chi-square tests (p < 0.01) using Prism software (GraphPad). FRET-FLIM experiments were repeated at least twice, and ANOVA was used to test statistical significance between different populations of data.

### FRET-FLIM Analysis and Superresolution Microscopy

Sixteen- to 20-hr-old embryos and 48-hr-old pupal wings were fixed with 4% formaldehyde, using standard procedures, for 20 min (embryos) or 2 hr (pupae) at room temperature. For FRET-FLIM, samples were incubated 15 min in NaBH_4_ (1 mg/ml in PBS) to reduce autofluorescence and mounted with FluorSave reagent (Calbiochem). Details of imaging FRET-FLIM and 3D-SIM are in Supplemental Experimental Procedures.

For each genotype analyzed by FLIM, n > 10 samples were imaged and only one image was analyzed per sample. All pixels within a single image were averaged to a single value, and the n values per genotype were used to calculate the mean FRET efficiency and SEM. Lifetime image examples shown are presented using a pseudocolor scale whereby blue depicts normal GFP lifetime (i.e., no FRET) and red depicts reduced GFP lifetime (areas of FRET). For each genotype analyzed by 3D-SIM, n > 5 samples were imaged and only one image was analyzed per sample.

## Author Contributions

B.K. performed all experiments except the FLIM imaging (Figures 5A–5D, 6A, and 6C), which M.P. advised on and performed. S.L.H. and N.H.B. performed the genetic screen. J.W. generated *βPS-GFP* and *Vinc-GFP/RFP*. S.L.H. and R.J. generated and mapped *ΔVinc*. N.H.B. generated the *GFP-talin* and *Δhead* constructs. B.K., S.L.H., and N.H.B. designed the experiments. B.K. and N.H.B. wrote the paper.

## Supplemental Information

Document S1. Supplemental Experimental Procedures, Figures S1–S4, and Table S1

Document S2. Article plus Supplemental Information
