# Supplementary material for: Alternative Mechanisms for Talin to Mediate Integrin Function
Source: Curr Biol. 2015 Mar 30;25(7):847–57. doi: 10.1016/j.cub.2015.01.043 (PMC4386027; doi:10.1016/j.cub.2015.01.043)
Supplement: Document S1. Supplemental Experimental Procedures, Figures S1–S4, and Table S1 [file mmc1.pdf]

Current Biology
Supplemental Information

Alternative Mechanisms for Talin to Mediate Integrin Function

Benjamin Klapholz, Samantha L. Herbert, Jutta Wellmann, Robert Johnson, Maddy Parsons, and Nicholas H. Brown

Supplemental Figures

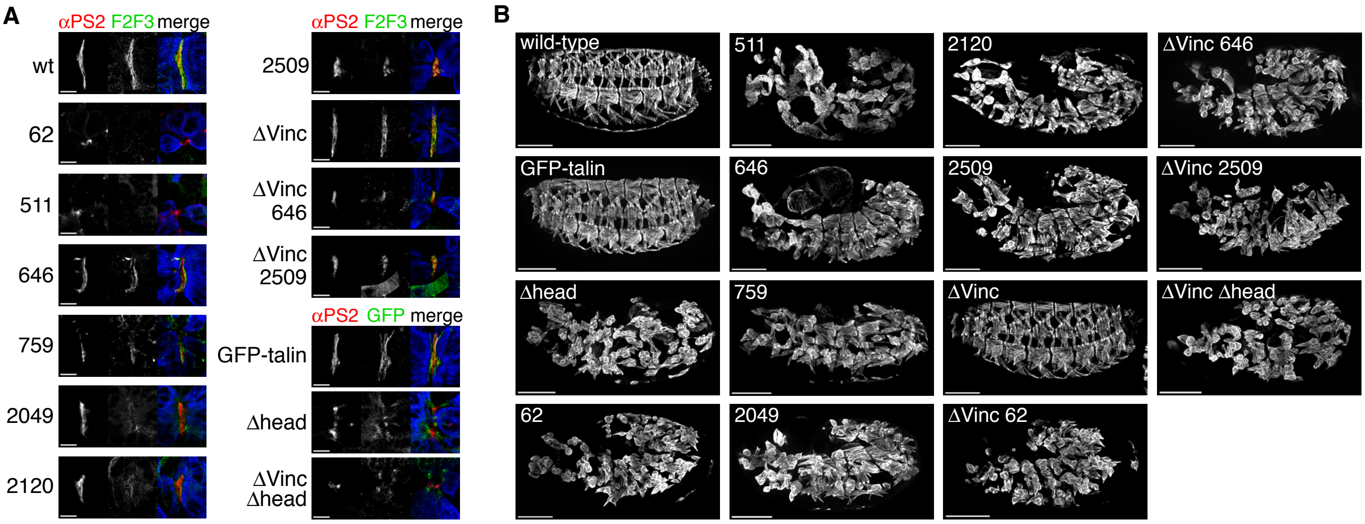

Figure S1, related to Figure 2.

Different mutant talins cause a range of muscle detachment phenotypes.

(A) Enlarged views of ventral-longitudinal muscle attachments stained for muscle myosin (blue), αPS2 integrin subunit (red) and talin F2-F3 domain (F2F3, green) or GFP (green) as indicated. Scale bars are 10μm.

(B) Muscle myosin staining of muscles of wild-type or maternal/zygotic mutant embryos, some also exhibit a germband retraction defect, e.g. talin62. Scale bars are 100μm.

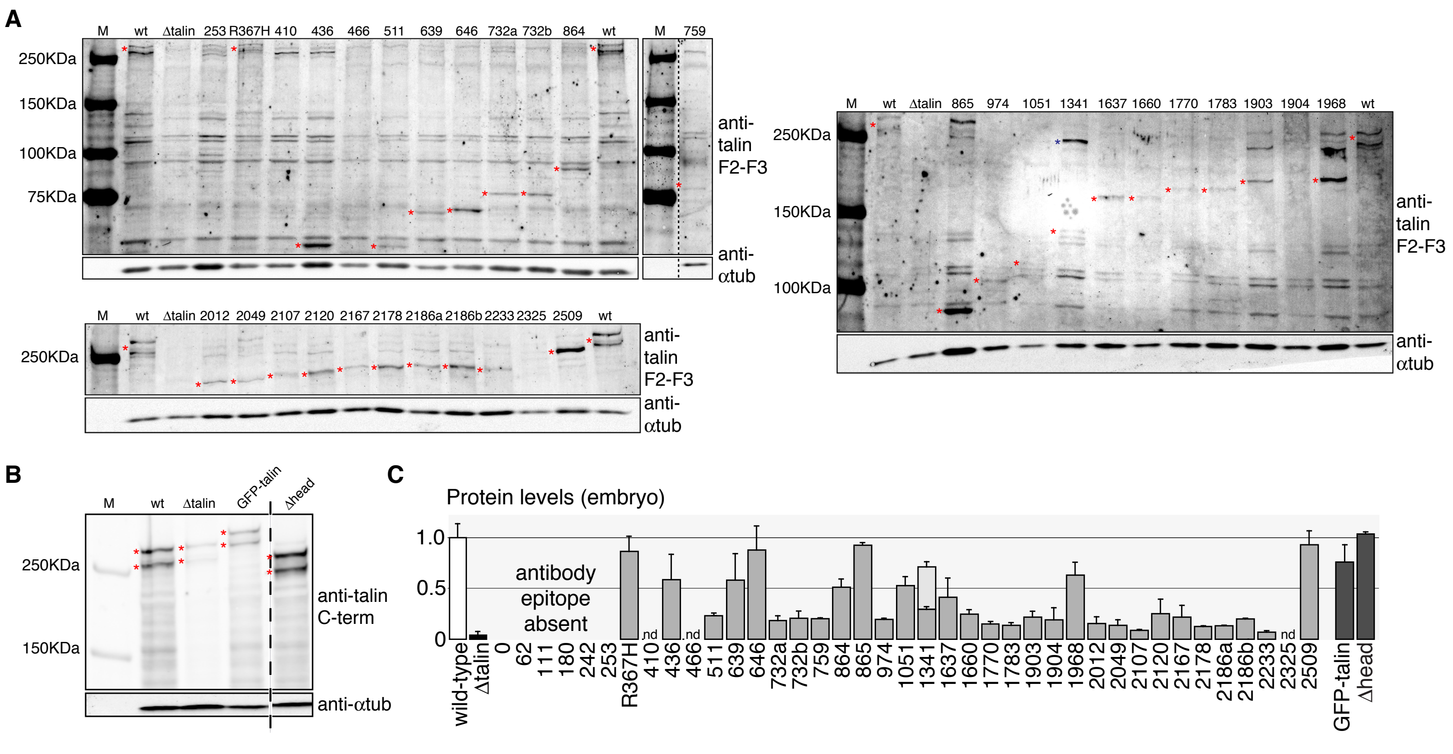

Figure S2, related to Figure 1.
Quantitation of mutant talin protein levels.
(A,B) Talin truncations (A), transgenes (B) and αtubulin (bottom panels) were detected in western blots of embryonic extracts of homozygous mutants. Talin was detected with anti-F2-F3 (A) or anti-C-terminus (B) antibodies. *Wild-type* (wt) and *Df(3L)79a* (Δtalin) embryos serve as controls. Red asterisks indicate the bands used for quantification in (C) and the blue asterisk in 1341 indicates the unexpected large protein in *rhea[C33]* embryos. The dashed line in (B) indicates a lane deleted from the picture of the blot.
(C) Levels of mutant talin in embryos homozygous for the point-mutations (light grey bars) or site-directed mutations (dark grey bars). Two sizes of talin were found in *rhea[C33]* (talin1341); the larger protein is in lighter grey. nd = not detectable. Standard deviations from two independent experiments are shown.

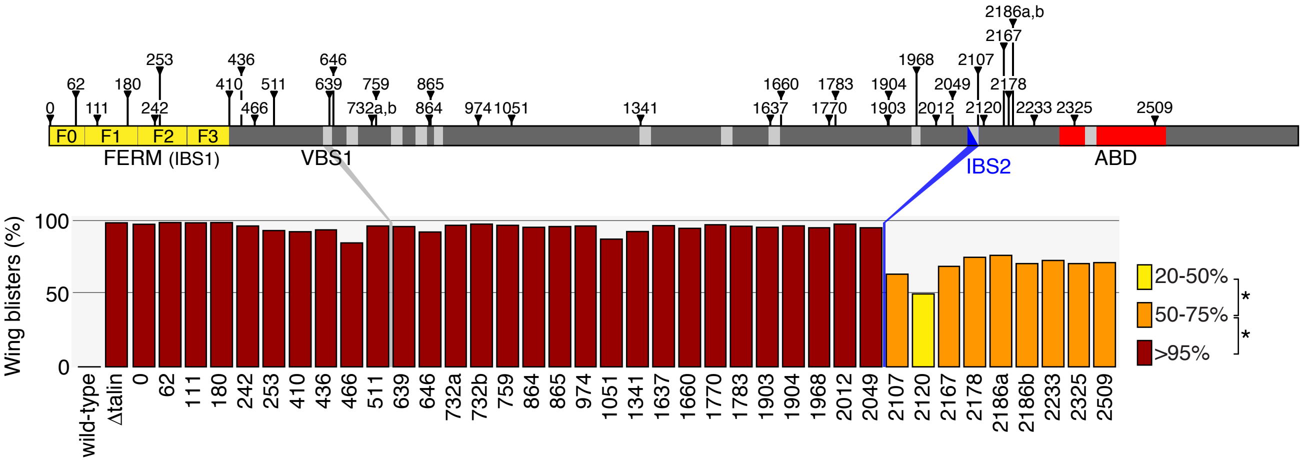

Figure S3, related to Figure 4.

Distribution of wing blister phenotypes in all 38 point mutants that truncate talin.

Quantification of wing blister frequency for all truncation mutants, from ≥100 flies/mutant. Bar colours show statistically distinct categories (*= $p<0.01$). The flat bar of wild-type indicates no defect.

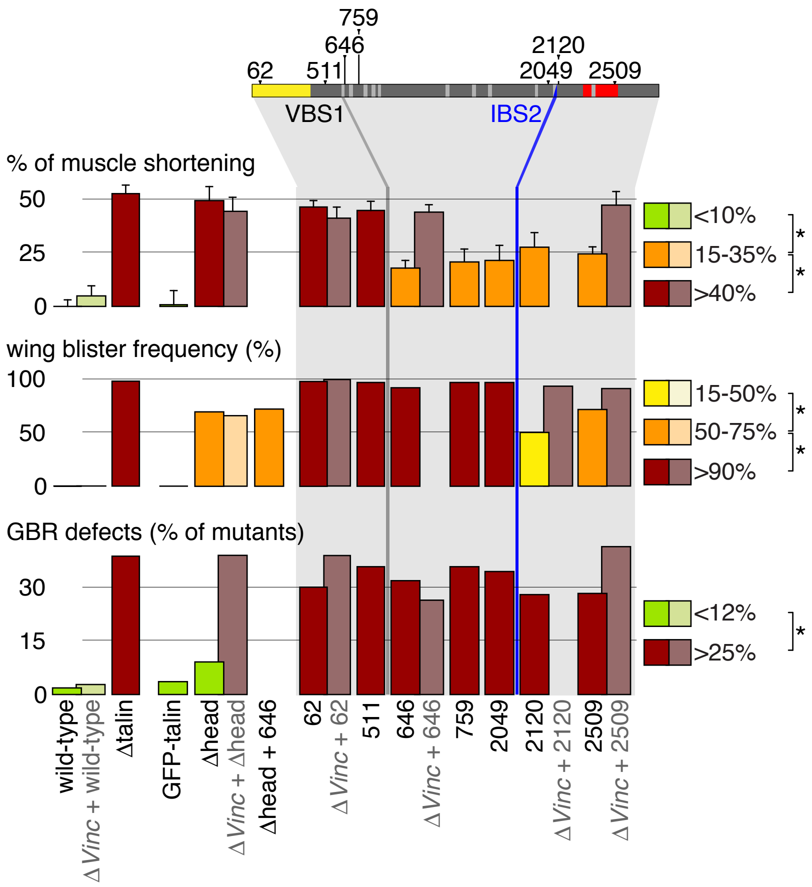

Figure S4, related to Figure 7.

Talin mutations have variable activities in three distinct integrin-mediated processes.

This figure summarises quantitation of the phenotypes caused by select talin mutations to permit comparison between tissues. The histograms are identical to those in Figures 2B, 3B and 4B. The lighter colour bars show defects in the absence of vinculin. Flat bars (horizontal lines) indicate no defect. Genotypes not analysed do not have a bar. Bar colours show statistically distinct categories (p<0.01, green is not significantly different from wild-type).

Talin protein	*rhea* allele	DNA sequence change	Mutation	Predicted protein
**0**	A75	*GCCAGG* AT>AG TCC ACA	M1>K	?
**62**	B28	GGG GTT TGG>A TTG GAA	W63>STOP	1-62
**111**	C160	GTG TCG C>TAG CTT ATG	Q112>STOP	1-111
**180**	B20	*G* TG AAC TG>AG GTG GAT	W181>STOP	1-180
**242**	C103	*ATTCTTTAG>A* CT TGC	acc of 4th exon G>A	1-242 + 13 out of frame
**253**	C19	CAT ATT C>TAA TTT GGA	Q254>STOP	1-253
**R367H**	C208	GTA CGC CG>AT TGG GGC	R367>H	2836, R367H
**410**	C69	ATT CTT A>TAG AAG AAG	K411>STOP	1-410
**436**	A52	CCA TCC A>TA *GTAAGTG*	K437>STOP	1-436
**466**	C92	*CGAAACATAG>A* GC GAG	acc of 7th exon G>A	1-466 + 3 out of frame
**511**	C130	CCA CAG C>TGA GCT CTG	R512>STOP	1-511
**639**	A80	CTC CTC A>TAG GCA GCC	K640>STOP	1-639
**646**	A109	GAA AGC A>TAG GAG CCG	K647>STOP	1-646
**732a**	C86	ACC AGT C>TAA TTG GTG	Q733>STOP	1-732
**732b**	C166	ACC AGT C>TAA TTG GTG	Q733>STOP	1-732
**759**	B128	GCT GCC A>TAA AAT GTG	K759>STOP	1-759
**864**	B33	GCG GAT C>TAG CAA CAG	Q865>STOP	1-864
**865**	B7	GAT CAG C>TAA CAG GAT	Q866>STOP	1-865
**974**	C89	GAT TGC A>TAG CGA GTG	K975>STOP	1-974
**1051**	A79	TCG GA [del] AGC GTA CAC	Δ301bp	1-1051 + 14 out of frame
**1341**	C33	AAT GC<u>G</u> A><u>TAG</u> <u>A</u>AC CTG	K1342>STOP	1-1341
**1637**	C190	GTG TGG C>TAA CAG CTC	Q1638>STOP	1-1637
**1660**	C102	CGC GAC A>TAG GCT CCT	K1661>STOP	1-1660
**1770**	C38	TGC GAA C>TAG GTG CTC	K1771>STOP	1-1770
**1783**	C59	CTG GTC C>TAA TCG GCC	Q1784>STOP	1-1783
**1903**	C90	AAC TAT C>TAA CAA TTG	Q1904>STOP	1-1903
**1904**	B150	TAT CAA C>TAA TTG ACT	Q1905>STOP	1-1904
**1968**	C18	TCC GAA A>TAG GTG GCC	K1969>STOP	1-1968
**2012**	B63	GGA ACT TT>AG CAT TCG	L2013>STOP	1-2012
**2049**	A16	GGA ACT C>TAA GAC CAG	Q2050>STOP	1-2049
**2107**	C197	TGC ACT A>TAG TTG GCC	K2108>STOP	1-2107
**2120**	C63	TCC ATG C>TAG GAT CTT	Q2121>STOP	1-2120
**2167**	A78	*TGGAATT[del]CCAG* GCC CCA	acc of 12th exon Δ27bp	1-2167 + 3 out of frame
**2178**	C193	AAC ACC C>TAG GTG GGA	Q2179>STOP	1-2178
**2186a**	C119	CTG ATC C>TGA GTG ACC	R2187>STOP	1-2186
**2186b**	C141	CTG ATC C>TGA GTG ACC	R2187>STOP	1-2186
**2233**	C206	GTG GCC TGG>A AAC TGT	W2234>STOP	1-2233
**2325**	C49	GCC GCC AAG [del] T CCT CGT	Δ17bp	1-2325 + 26 out of frame
**2509**	C110	AAC GCT C>TGA TCC GCC	R2510>STOP	1-2509

**GFP-talin**	GFP-talin	No sequence change	Wild-type talin	(GFP)1-2837
**GFP-Δhead**	GFP-talinΔhead	**[GFP]** [del] CTA AAT GTG GAG	Δ450aa in N-terminus	(GFP)451-2837

Table S1, related to Figure 1.

Molecular description of the 39 new alleles of *rhea (talin)* and site-directed talin mutants.

Protein names were determined according to the last in-frame residue encoded for each mutant allele. In DNA sequence change column, the change is shaded grey, non-coding sequence is in italics, and the new splice donor in allele $rhea^{C33}$ is underlined.

Supplemental Experimental Procedures

Generation of new mutant alleles of rhea (talin) and Vinculin

The new alleles of *rhea (talin)* were isolated in a genetic screen for mutations on chromosome arm 3L that cause wing blisters when homozygous mutant clones were generated in the wing. The 3L arm contains four known wing blister loci: *rhea(talin), Fermitin1 (Fit1), Fermitin 2(Fit2) and Integrin-linked-kinase.* The Fermitins are *Drosophila* orthologues of kindlins; *Fit1* mutations are lethal while those in *Fit2* are viable, and only clones mutant for both *Fermitins* produce wing blisters (our unpublished results). Therefore, to be able to recover new *Fit1* mutations in the screen (to be described elsewhere), we started with flies containing the viable null allele *Fit2[83]* so that double mutants could be generated. While this was useful for our screen it has no impact on the results described here. We tested whether the absence of Fit2 had any effect on the *rhea* mutant phenotypes described in this work, by comparing the phenotype of key alleles in the presence of a *Fit2* rescue construct or when the *Fit2[83]* was recombined off, and found that there was no effect caused by the lack of Fit2 (data not shown).

The efficiency of previous screens [S1, S2] was improved in two ways: by switching from X-rays to the more efficient chemical mutagen EMS and by restricting recombination events to the wing by driving FLP recombinase with *vestigial-Gal4* [S3] rather than the heat shock induced FLP expression. Thus, ethyl methanesulfonate mutagenised w/Y; *Fit2[83] P{FRTw[hs]}2A* males were crossed to *w; P{w[+], Gal4}Vg[BE] P{w[+], UAS::FLP}/ CyO; P{FRT}2A* (with the white+ excised from *P{FRT2Aw[hs]}*) females, and w/Y; *P{w[+], Gal4}Vg[BE] P{w[+], UAS::FLP}; * Fit2[83] P{FRTw[hs]}2A/P{FRT}2A* males with wing blisters were selected, retested and balanced. The confirmed wing blister mutants were then tested for allelism with *rhea*. From a series of three screens we isolated 39 new mutant alleles of *rhea* from about 50,000 F1 individuals. For an amorphic allele of talin (Δtalin) we used the stock *w[*]; Df(3L)79a P{FRTw[hs]}2A* [S4]. To identify the sequence changes in the new *rhea* alleles, we sequenced from the N-terminus and stopped sequencing a particular

allele when we encountered a stop codon or frameshift; for *rhea[A75]* and *rhea[C208]* (encoding $talin^0$ and $talin^{R367H}$), we sequenced the entire coding region. Deletions in the *Vinculin* gene were obtained by imprecise excision of the GenExel EP element *G1390*. *Df(1)Vinc[2] (ΔVinc)* deletes 20-30 Kb (mapped by PCR) which include the whole *Vinculin* coding sequence as well as the *msta* gene and part of the *Mct1* gene.

Generation of genes expressing fluorescently tagged talin, vinculin and βPS integrin subunit

The *GFP-talin* construct contains a 30,275 bp fragment comprising the 16,284 bp 5' of the *rhea* ATG initiating codon, the GFP-coding sequence, a 4 serine linker, the rest of the *rhea* gene extending to 925 bp 3' of the stop codon. The *GFP-Δhead* construct contains the same DNA sequence as *GFP-talin* but with deletion of residues 1-450. Two insertions for each rescue construct were tested and had indistinguishable rescue activity. The *Vinculin-GFP* and *Vinculin-RFP* constructs contain a 19,979 bp fragment extending from 6,266 bp 5' of the *Vinculin* ATG initiating codon to 7,495 bp 3' of the stop codon with a 4-serine linker and the GFP or RFP sequence inserted after the last codon. These DNA constructs were cloned in the transposable P-element vector *pWhiteRhino* for transgenesis (NHB unpublished). βPS-GFP was generated by "ends-out targeting" homologous recombination [S5], inserting a 4-serine linker followed by the *GFP* sequence after the last codon of the *myospheroid* (βPS) gene. Recombinants were selected by fluorescence at MASs.

Western blot assays

Western blots were performed on twenty homozygous or heterozygous stage 17 mutant embryos, or ten 96-hr old pupal wings, for each genotype tested. Samples were lysed (50 mM Tris pH8, 150 mM NaCl, 0.5% Triton X-100, 1 mM $MgCl_2$, 0.1 mM EDTA, Proteinase inhibitor mix (Roche Complete EDTA-free, 1 tablet/25ml), mixed 1:1 with 4X Laemmli buffer, centrifuged 3 min at maximum speed, denatured 5 min at 95 ˚C and loaded on a SDS-PAGE 7.5% (for $talin^{253}$ to $talin^{864}$) or 6% (for $talin^{865}$ to $talin^{2509}$) gel. Western blots were carried out

according to standard procedure. Proteins were transferred to a PVDF membrane (Immobilon-FL, Millipore), the blocking solution was PBS, 0.1% Tween-20 and 50% blocking buffer (Odyssey), the primary antibodies were rabbit anti-talin F2-F3 domain [S6] (1:200), rabbit anti-talin C-terminus [S4] (1:1000) and mouse anti-αtubulin (1:5000, DM1A). The secondary antibodies were goat anti-mouse IRDye 800cw (1:10000, Odyssey) and goat anti-rabbit Alexa Fluor 680 (1:10000, Invitrogen). The blots were scanned with a LI-COR (Odyssey) scanner and the images were analysed with ImageJ. Talin amounts were determined by normalizing the talin staining to the α-tubulin staining, after subtracting background levels.

FRET-FLIM and super-resolution microscopy

Multiphoton, time-correlated single-photon counting (TCSPC) FLIM was performed to quantify interactions between defined protein pairs by FRET as described previously [S7]. Briefly: a Nikon TE2000E inverted microscope combined with an in-house scanner and Chameleon Ti:Sapphire ultrafast pulsed multiphoton laser (Coherent) was used for excitation of GFP (at 890 nm). All images were acquired to provide enough photon arrival times to enable accurate fitting of fluorescence decay while avoiding detector pile-up. Fluorescence lifetime imaging capability was provided by time-correlated single photon counting electronics (Becker & Hickl, SPC 700). Data was analysed by performing a single-exponential pixel fit in TRI2 time-resolved image analysis software (developed by Dr. Paul Barber, Gray Institute, Oxford, UK).

3D-SIM was performed using a DeltaVision OMX BLAZE V3 (Applied Precision), a 3D-SIM [S8] system equipped with sCMOS cameras. All data capture used an Olympus 60x 1.4NA oil objective, 488 nm and 593 nm laser illumination and standard excitation and emission filter sets. 3D-SIM image stacks were sectioned using a 125 nm Z-step size. Raw data was reconstructed using softWoRx 6.0 (Applied Precision) software.

Supplemental References

S1. Prout, M., Damania, Z., Soong, J., Fristrom, D., and Fristrom, J.W. (1997). Autosomal Mutations Affecting Adhesion Between Wing Surfaces in Drosophila melanogaster. Genetics *146*, 275-285.

S2. Walsh, E.P., and Brown, N.H. (1998). A Screen to Identify Drosophila Genes Required for Integrin-Mediated Adhesion. Genetics *150*, 791-805.

S3. Vegh, M., and Basler, K. (2003). A Genetic Screen for Hedgehog Targets Involved in the Maintenance of the Drosophila Anteroposterior Compartment Boundary. Genetics *163*, 1427-1438.

S4. Brown, N.H., Gregory, S.L., Rickoll, W.L., Fessler, L.I., Prout, M., White, R.A.H., and Fristrom, J.W. (2002). Talin Is Essential for Integrin Function in Drosophila. Dev Cell *3*, 569-579.

S5. Rong, Y.S., and Golic, K.G. (2000). Gene targeting by homologous recombination in Drosophila. Science *288*, 2013-2018.

S6. Tanentzapf, G., Martin-Bermudo, M.D., Hicks, M.S., and Brown, N.H. (2006). Multiple factors contribute to integrin-talin interactions in vivo. J Cell Sci *119*, 1632-1644.

S7. Parsons, M., Messent, A.J., Humphries, J.D., Deakin, N.O., and Humphries, M.J. (2008). Quantification of integrin receptor agonism by fluorescence lifetime imaging. J Cell Sci *121*, 265-271.

S8. Schermelleh, L., Carlton, P.M., Haase, S., Shao, L., Winoto, L., Kner, P., Burke, B., Cardoso, M.C., Agard, D.A., Gustafsson, M.G., et al. (2008). Subdiffraction multicolor imaging of the nuclear periphery with 3D structured illumination microscopy. Science *320*, 1332-1336.
